# Enabling the Polymer Circular Economy: Innovations in Photoluminescent Labeling of Plastic Waste for Enhanced Sorting

**DOI:** 10.1021/acspolymersau.2c00040

**Published:** 2022-12-12

**Authors:** Ryan R. Larder, Fiona L. Hatton

**Affiliations:** † Department of Materials, Loughborough University, Loughborough LE11 3TU, United Kingdom

**Keywords:** plastic waste, recycling, plastic sorting, circular economy, photoluminescent markers, tracer-based sorting, sustainable packaging, waste management, fluorescent markers

## Abstract

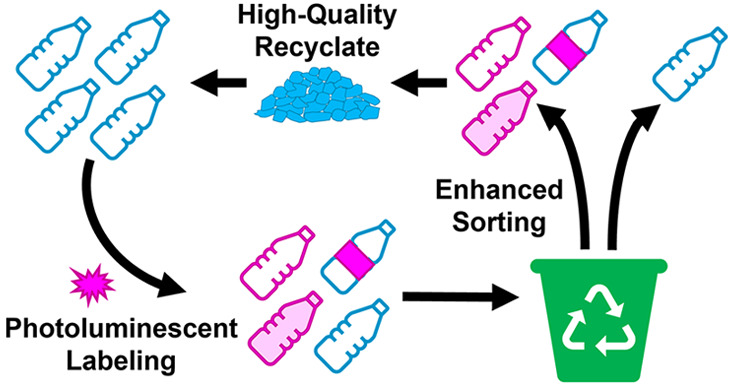

It is widely accepted that moving from a linear to circular economy for plastics will be beneficial to reduce plastic pollution in our environment and to prevent loss of material value. However, challenges within the sorting of plastic waste often lead to contaminated waste streams that can devalue recyclates and hinder reprocessing. Therefore, the improvement of the sorting of plastic waste can lead to dramatic improvements in recyclate quality and enable circularity for plastics. Here, we discuss current sorting methods for plastic waste and review labeling techniques to enable enhanced sorting of plastic recyclates. Photoluminescent-based labeling is discussed in detail, including UV–vis organic and inorganic photoluminescent markers, infrared up-conversion, and X-ray fluorescent markers. Methods of incorporating labels within packaging, such as extrusion, surface coatings, and incorporation within external labels are also discussed. Additionally, we highlight some practical models for implementing some of the sorting techniques and provide an outlook for this growing field of research.

## 1 Introduction

Plastic waste represents a huge global challenge with over 350 million tons of plastic produced worldwide annually; however, the end-of-life considerations for these plastics are rarely considered. The estimated global recycling rate of all mass-produced plastics was 9% as of 2015^1^ and varies greatly depending on region. For example, in the US, the rate of plastic recycling was estimated to be 8.7% in 2018,^2^ whereas in Europe, around 35% of postconsumer plastic waste was sent to recycling facilities while 42% was sent to energy recovery facilities and 23% was sent to landfill (in 2020).^3^ While data on plastics recycling is more widely reported and known for developed countries, they are less reported for developing countries. Data on the recycling of municipal solid waste in developing countries, including plastics, varies significantly, whereby stakeholder engagement and collaboration was identified as a major influence.^4^ Moreover, in developed countries with collection and sorting infrastructure, the recovery of plastics is likely to exceed utilization, whereas the opposite is more likely in developing countries.^5^

Plastic packaging constitutes the largest sector in plastic demand, where polyethylene (PE) (high and low density), polypropylene (PP), polyethylene terephthalate (PET) and polystyrene (PS) are commonly used commodity thermoplastics.^3^ As such, this sector contributes a large amount of plastic waste annually, and 95% of the plastic packaging material value is lost because it is typically single-use.^6^ While efforts focus on shifting from linear to circular economies for plastics, emphasis is placed on reduction, reuse, and recycling, identified as beneficial waste management strategies compared with landfill and mismanaged disposal.^7^ In the food packaging industry, reduction of the use of plastic in packaging can be achieved by either avoiding excessive packaging or overpackaging or by reducing the weight and thickness of the packaging without compromising functionality.^8^ Both of these approaches reduce processing, transportation, and distribution costs; reduce associated CO_2_ emissions; and improve sustainability. Life cycle assessment of carbonated drinks packaging associated between 59 and 77% of environmental impacts with the packaging itself rather than the ingredients, manufacturing, and processing or transportation.^9^ For PET bottles, their global warming potential could be greatly reduced (by 32–48%) if the recycling rate of PET reached 40–60%.^9^

Reuse is currently possible in some areas, mainly through trial schemes at local levels, and is proposed as a solution to move toward a circular economy and reduce single-use plastics for food packaging.^10−12^ However, in principle, plastics are excellent candidates for recycling, which offers flexibility when compared with reuse because products can be reprocessed into different shaped objects. Current hurdles to the efficient recycling of plastic packaging are collection and sorting to give high-quality waste streams that can be used in reprocessing, as cross-contamination between different commodity thermoplastics can lead to issues when reprocessing to produce new products. Moreover, with increasing societal pressure for packaging with increased recycled content, governments have introduced legislation to drive change in this area. In the UK, the recent Plastic Packaging Tax legislation (2022) enforces businesses to pay tax on any manufactured or imported packaging that does not meet the minimum requirement of 30% recycled content by weight, thereby encouraging businesses to use recycled, rather than virgin, plastics.^13^ New legislation in the UK, “Extended Producer Responsibility,” will also require producers to bear costs for managing packaging waste from households and street bins in attempts to reduce plastic waste and promote a circular plastics economy.^14^ Within Europe, the European strategy for plastics was adopted in January 2018 with aims to “support more sustainable and safer consumption and production patterns of plastics,”^15^ for example, by introducing new rules for businesses to encourage increased recyclability of plastics. Across the globe, over 60 countries have introduced bans and levies to target plastic packaging (plastic bags and Styrofoam).^16^ Therefore, because of societal pressures and legislative changes, considerable efforts have been placed on improving current recycling capabilities, including enhanced detection and sorting of plastic packaging.^17^

**Figure 1:**
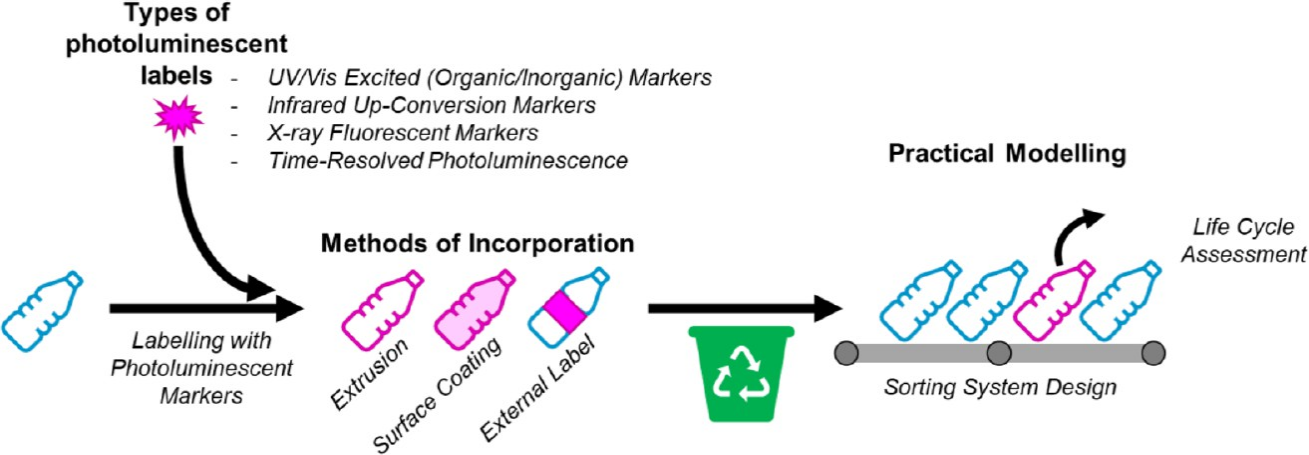
Schematic overview of inclusion of photoluminescent markers into plastic articles, highlighting key areas for consideration.

Herein, we discuss the current methods of plastic sorting and review techniques for labeling packaging to (i) aid the detection and sorting of postconsumer plastic packaging waste, (ii) improve recycling/reuse rates, and (iii) generate high-quality reusable plastic feedstocks to enable global circular polymer use. Current innovations in packaging technology, in particular enhanced labeling, are reviewed, including the current state of the art in plastics sorting; applications of advanced labels, such as UV–vis, infrared, and X-ray photoluminescent markers; and time-resolved photoluminescent labels and digital labels. Methods of incorporating advanced labels in packaging are discussed, and existing practical models are highlighted, including life cycle assessment, with conclusions and an outlook on how current “smart” packaging technologies can be utilized to specifically enable the circularity of packaging (Figure 1).

## 2 Current Methods of Plastic Sorting

Globally, current approaches to recycling can vary significantly depending on various socioeconomic factors. In more developed countries, infrastructure is established to collect and sort waste at materials recycling facilities. However, in developing countries, this is not always the case, and plastics are generally disposed of in the environment or in landfill. For the purposes of the discussion here, we focus on establishing materials recycling facilities that exist in developed countries.

**Figure 2:**
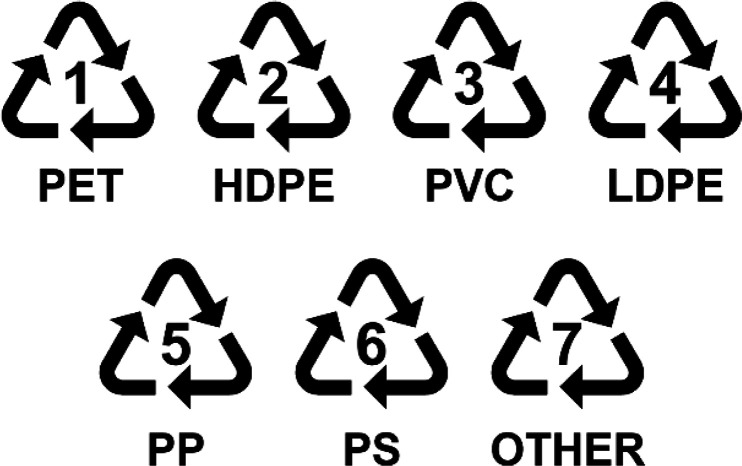
Plastic resin code labeling system.

Depending on the local infrastructure, plastics may need to be separate from nonplastic recyclates (i.e., metals, cardboard, paper, glass), if collected with mixed recycling,^18,19^ or they may be sorted during the collection process, e.g., bottle return schemes. Once isolated, the separation of various types of plastic waste is most commonly performed using a combination of manual sorting,^20^ sink-float,^21^ froth flotation,^22^ infrared (IR) spectroscopy, and optical color recognition sorting.^18,23^ Manual sorting is dependent on an understanding of which types of plastic are typically used for different types of product or packaging, and many commodity plastics also bear a symbol indicating the plastic type (see Figure 2). This is a time-consuming, labor intensive, and expensive method because of the need to employ workers to carry out the sorting.^20^

Flotation relies on the difference in densities between different plastics, whereby plastics with lower densities than water (1.0 g cm^–3^) will float, e.g., PE and PP with densities ∼0.9 g cm^–3^, while plastics with densities greater than water will sink, such as PET and polyvinyl chloride (PVC) with densities of ∼1.4 g cm^–3^, to enable a simple method of sorting.^18,21^ Sorting techniques based on IR spectroscopy are usually automated. Typically, plastics are transported by conveyor belt, and an infrared detector is used to identify various plastics before separation using jets of air.^18,19^ While IR sorting is highly automated, there are several limitations, including when colored plastics are used, especially black plastics, because the irradiated light is absorbed.^24^ Other limitations include the sorting of composite materials and contaminated plastics, which present challenges because they can prevent the IR detection from successfully identifying the plastic.

Also, plastics that have been in contact with hazardous materials represent a significant problem because these must be removed if the sorted plastic is intended to be recycled and used for food-contact applications.^25^ None of the currently used automated methods can identify and remove these contaminated materials. Other methods of detection, such as the use of UV–vis, IR, and X-ray photoluminescent markers and digital markers, have been investigated extensively to overcome these limitations. Here, we review the current state of the art and future outlook for the photoluminescent labeling of packing to enable more efficient sorting.

## 3 Types of Photoluminescent Labels

Molecules that exhibit photoluminescence are a popular candidate for identification of specific plastic waste streams. This is not an overly new concept, with the idea first put forward by Luttermann et al. in a patent filed in 1990, however, recent global developments have now renewed its interest.^26^ The unique emission wavelengths of particular chemical compounds provide a robust method for identification and separation of waste plastics, without interfering with existing sorting techniques. Photoluminescent labels can be broadly categorized by their excitation source, either by UV–vis, infrared or X-ray radiation. A summary of the general summary of the characteristics of each marker type is provided in Table 1 and discussed in detail in this section.

**Table 1:** Assessment of the Different Types of Photoluminescent Markers That Can Be Applied to Label Polymers

type of marker	UV–vis		IR (up-conversion)	X-ray	time-resolved auto-fluorescence
	organic markers	inorganic markers			
**minimum quantity**	**low**	**high**	**low**	**high**	**none**
What minimum concentration of marker is required for detection?	0.1 ppm (Langhals et al.)^27^	0.1 wt % (Massardier et al.)^29^	10 ppm (Woidasky et al.)^31^	0.1 wt % (Bezati et al.)^32^	
	10 ppm (Arenas-Vivo et al.)^28^	1 wt % (Becker et al.)^30^			
**emission range**	∼400–650 nm^27,28,33−35^	285–650 nm^29,30,36^	500–700 nm^31^	5–60 keV^37^ (0.021–0.248 nm)	∼400–500 nm^38^ (dependent on polymer)
What is the emission wavelength?					
**lifetime**	**short**	**long**	**likely long**	**very long**	**possibly unlimited**
How long can the marker be detected for?	Organic compounds degraded by thermal and photochemical degradation.	Less susceptible to degradation processes.	Inorganic makeup gives good stability, though the effect of lifetime on up-conversion efficiency is not known.	Detection only requires the presence of atomic elements instead of compounds.	Lifetime inherently linked to the polymer; not known if polymer degradation will impede detection.
**impact**	For HDPE, ***no change*** to thermal properties at loading of 10 ppm.^28^	For PP, a small increase in crystallinity, Young’s modulus, and impact strength was observed at loading of 0.1 wt %.^29^	not yet tested	For PP, ***no change*** in thermal or crystallization properties at 1 wt % marker loading; *small* reduction of elongation at break at 0.1 wt % loading.^39^	none
What is the impact on polymer properties?	No testing of mechanical properties, but concentrations are low enough to assume this is negligible.				
**impurity tolerance**	**low**	**medium**	**high**	**high**	**low**
How well can the markers be detected in the presence of foreign materials?	Highly susceptible to interference from other organic matter.	Longer phosphorescence emission times can be used to distinguish from fluorescence from impurities.	Up-conversion photoluminescence is uncommon in most materials.	Only the predetermined element needs to be detected.	Photoluminescent lifetime of foreign components may severely limit detection.
**technological demand**	**medium**	**medium**	**low or high**	**high**	**possibly high**
How significant is the required change in sorting infrastructure to adopt these markers?	Requires incorporation of relatively straightforward UV–vis light sources and optical detectors.	Requires incorporation of relatively straightforward UV–vis light sources and optical detectors.	Low demand if emission can be induced with existing IR light sources; high, otherwise, as this will require use of lasers for sufficient excitation.	Requires use of costly X-ray sources and detection of high-energy photons.	Detection is based on analysis of photoluminescent lifetime rather than emission wavelength; this may require more sophisticated detectors.

It should be noted that the process of photoluminescence can be defined as either fluorescence or phosphorescence, depending on the radiative emission pathway. However, the term fluorescence is often, and occasionally incorrectly, used in the literature to describe any form of photoluminescence. Here, we attempt to use this terminology as accurately as possible.

### 3.1 UV–Vis Photoluminescent Markers

The most common excitation source to induce photoluminescence in chemical compounds is light in the UV–vis region, as the energy of these photons relate most closely to that of the electron energy levels in molecules. Many molecules can be excited by UV or short wavelength visible light and then emit the absorbed radiation in a variety of visible colors, making detection reasonably trivial. The extensive range of chemical candidates and ease of detection make UV photoluminescent markers the most popular for study in advanced labeling of plastic waste.^40^

**Figure 3:**
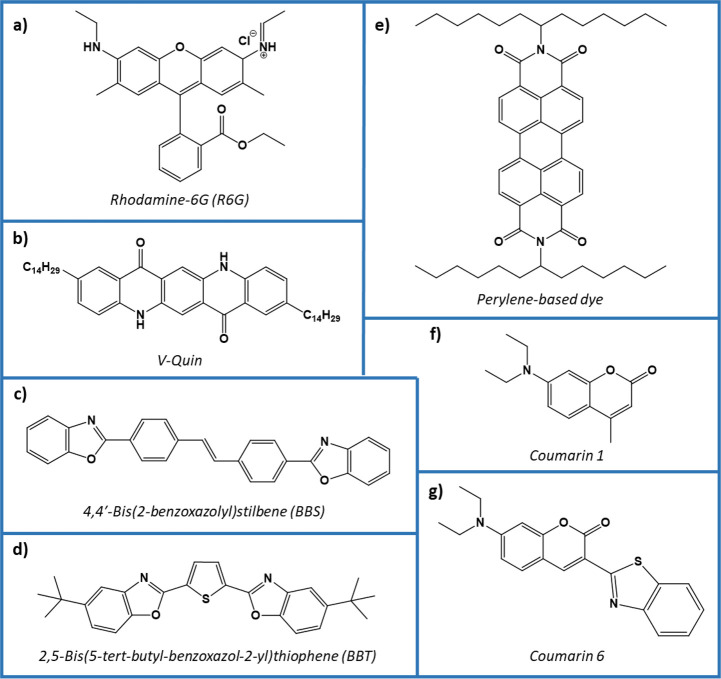
Chemical structures of organic fluorescent dyes used by (a,b) Arenas-Vivo et al.,^28^ (c,d) Pilon et al.,^33^ (e) Langhals et al.^27^ and (f,g) Müssig et al.^34^ to label commodity plastics discussed in this review.

UV–vis photoluminescent markers investigated for tracer-based sorting of plastics can be separated into organic and inorganic chemical compounds. Here, inorganic markers are generally defined as any compound containing metallic elements, such as metal oxides or metal complexes. Each class of compounds carry with them general advantages and disadvantages (Table 2), though there can be exceptions in each case. Commonly, organic compounds (Figure 3) have higher photoluminescent quantum yields, meaning smaller quantities can be employed while being above detection limits.^41^ However, inorganic compounds tend to be less susceptible to breakdown and degradation under UV exposure, meaning their detection will be more long-lived between production and waste collection life cycle stages. Inorganic compounds also tend to have longer photoluminescent lifetimes, as their emission processes tend to be phosphorescent rather than fluorescent, and slightly larger Stokes shifts, which opens up more detection solutions.^41^

**Table 2:** General Photoluminescent Properties of Organic and Inorganic Species^a^

	organic	inorganic
	e.g. perylenes, coumarins, rhodamines, and quinolines.	e.g. metal complexes and rare earth metal oxides.
photoluminescent quantum yield	variable, but generally high; highly dependent upon the solvent/environment	variable, but generally quite low
molar absorbance	variable, but generally high	variable, depending on ligand structure
emission spectra	broad, around 40–70 nm half-width	narrow, around 10 nm half-width
stokes shift	variable, commonly around 10–100 nm	quite large, around 100–200 nm
photoluminescent lifetime	short, around 0.5–5 ns	long, can be up to 1 ms
photostability	variable, but generally poor	relatively high
toxicity	variable, but often low	less definitive data available, can be quite high

a Adapted with permission from ref (41). Copyright 2009 Springer.

#### 3.1.1 Organic Fluorescent Markers

There are a huge number of existing small-molecule organic fluorophores that can be employed in plastic labeling. These fluorophores typically comprise aromatic components or cyclic or planar structures containing π-bonding. They can be grouped based on these core structural components, such as coumarins, xanthenes, benzoxadiazoles, and pyrenes to name a few.^42^ Only a relatively small number of these compounds have been investigated for use in the fluorescent labeling of commodity plastics.

Arenas-Vivo et al. investigated the use of two organic compounds as UV fluorescence tracers in high-density polyethylene (HDPE) as a method of identifying and removing heavily contaminated items not suitable for mechanical recycling.^28^ They investigated the use of two organic fluorophores, where the first was the well-known fluorescent dye Rhodamine-6G (R6G) (Figure 3a), and the second was a lab-made quinacridone derivative, V-Quin (Figure 3b). The key feature of this custom dye was the inclusion of long aliphatic C_14_ groups onto the quinacridone group, which improved its compatibility with the PE chains. Additionally, the R6G was tested for its compatibility with HDPE in both its neat form and when supported on a montmorillonite clay.

When compounded into HDPE samples, both dye markers were shown to significantly alter the optical emission spectrum, with additional peaks observed in the 550–650 nm range. It should also be noted that this change was discernible when comparing samples to HDPE containing both a small amount of CaCO_3_ and a phenol-phosphite compound, both common additives in commercial HDPE production. Incorporation of the dyes was additionally shown to have no effect on the crystallinity and thermal properties of the HDPE.

**Figure 4:**
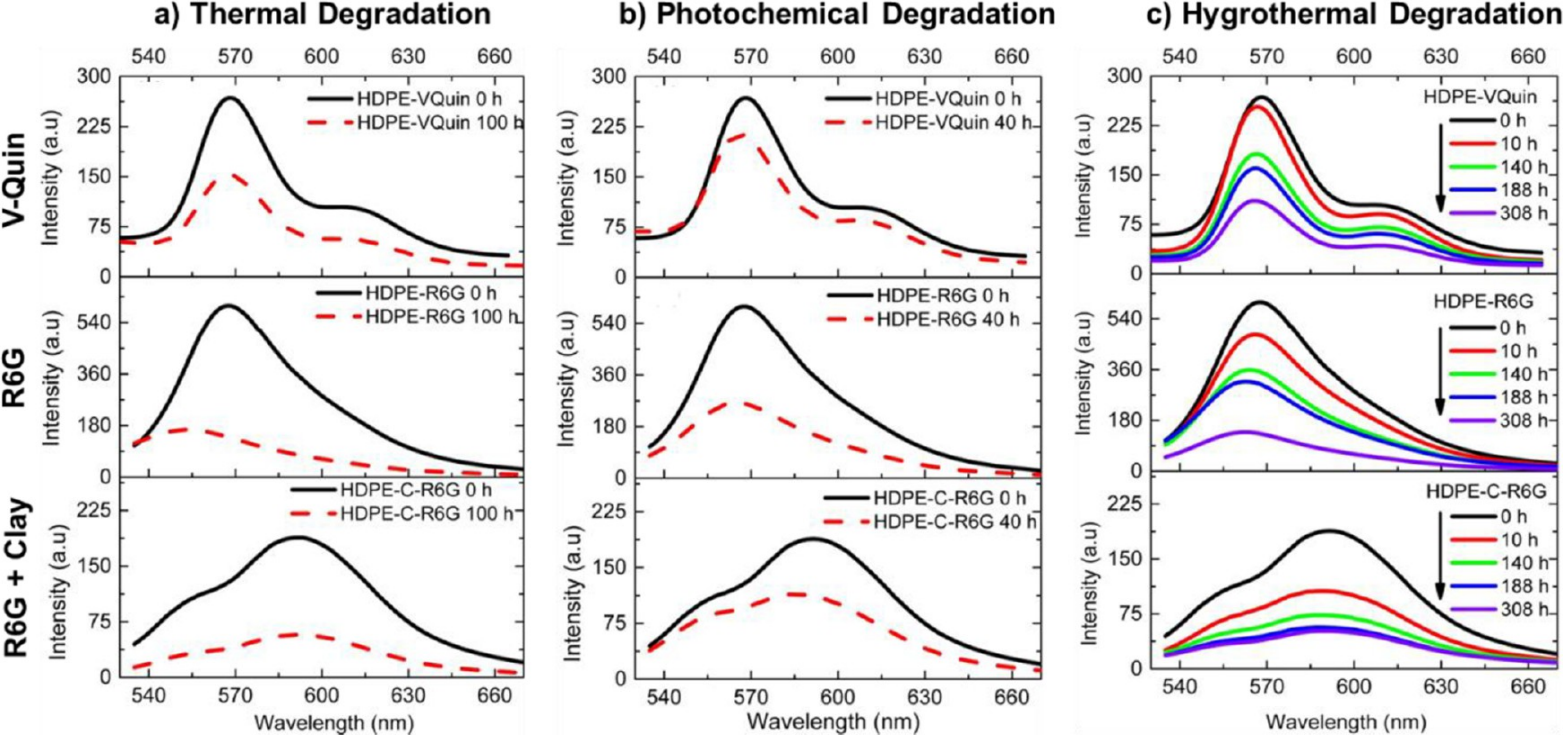
Fluorescent spectra demonstrating the impact of (a) thermal, (b) photochemical, and (c) hygrothermal degradation on HDPE embedded with the V-Quin dye (top row), R6G dye (middle row), and R6G dye along with a montmorillonite clay. For all V-Quin spectra, λ_ex_ = 350 nm, and for all R6G spectra, λ_ex_ = 510 nm. Reproduced with permission from ref (28). Copyright 2017 Elsevier.

Furthermore, Arenas-Vivo et al. subjected their labeled HDPE to accelerated aging studies to determine the longevity of their labeling; one of the only literature reports to do so.^28^ After simulated thermal (80 °C, 40 h), hygrothermal (deionized water, 80 °C, 308 h), and photochemical (UVB exposure, 100 h) degradation processes, the fluorescent intensity of all marked samples was shown to decrease (Figure 4). However, this decrease was significantly less for the HDPE marked with the V-Quin dye, with the characteristic peaks still clearly visible after all three degradation procedures. The authors attributed this to the anchoring of the V-Quin dye within the HDPE matrix, particularly its reduced lixiviation in the hygrothermal degradation. Though this may have implications for long-term use, the marker stability may still be applicable to short-term applications, such as packaging. The exact stability requirements for photoluminescent sorting labels in any application are yet to be defined, and as such, there is no benchmark to assess the success of these results.

A report by Langhals et al. demonstrates the use of a different class of UV organic fluorescence markers: perylene dyes (Figure 3e).^27^ This type of dye was selected for their very high quantum yields (close to 100%),^43^ but the authors also reference a key advantage of organic molecules, in general, is their good compromise between long-term stability and degradability. This, in theory, allows them to maintain visibility throughout the lifetime of a plastic product’s use but prevents the accumulation of dye additives in future waste streams, unlike many inorganic dye equivalents. In similarity to the V-Quin molecule, some of the perylene dyes investigated were modified to include long C_13_ chain aliphatic groups to aid in solubility into polymer matrices. Only polyoxymethylene (POM) was studied for compatibility with these dyes; however, it is reasonable to assume this additional solubility would also apply to common polyolefin polymers.

A range of perylene-based dyes were studied as fluorescent markers, each modified with chemical functionalities designed to shift the optical emission wavelength of the marker.^27^ Four dyes were presented with various emission peaks in the 500–800 nm visible region. Because each dye is discernible in a combined emission spectrum, this enables the use of a mixture of the perylene compounds for the binary encoding of plastic components, thereby allowing the separation of multiple waste streams. The authors argue that by also varying the concentration of each dye, an encoding system can be created to distinguish between up to 240 different items. However, as the emission wavelength of each dye is lowered by chemical modification, so too is the wavelength of excitation shifted further into the visible region. This variability in the wavelength of absorbed light may preclude certain dyes from being detectable in specific applications where additives are added to plastic components, such as pigments.

The use of organic fluorescent dyes to identify and sort plastic waste streams was also investigated in a European Commission funded project known as Polymark.^44^ An interim report from the project details their use of two fluorescent dyes, 4,4′-bis(2-benzoxazolyl)stilbene (BBS) (Figure 3c) and 2,5-bis(5-*tert*-butyl-benzoxazol-2-yl)thiophene (BBT) (Figure 3d), in labeling PET bottles.^33^ Both dyes were selected because their optical emission occurred at <450 nm when irradiated with UV light (365 nm), therefore not overlapping with the natural fluorescence of PET seen at around 400 nm. The other key advantage underpinning their selection is the fact that both dyes are additives approved for food contact by the FDA.^45^ This is especially important for application in PET materials, which are used extensively in food packaging. Neither dye was chemically modified for increased compatibility with the PET and they were instead only investigated for use as an external coating (see Section 4.2).

There are also some similar topics of research that prove relevant when attempting to identify a suitable organic fluorescent dye for polymer labeling purposes. One such example is in anticounterfeiting, where unique optical markers are developed to distinguish specific articles.^46^ In one example from Müssig et al., a synthetic route to produce dual magnetic and luminescent supraparticles (particles obtained from the assembly of smaller colloidal particles) is presented as a tool to help encode manufactured products.^34^ This is primarily designed as a security feature for implementation into small articles as an invisible marker that can be scanned to confirm the legitimacy of manufacture. However, one can easily see how such a technology could also be applied as labels for the sorting of plastics.

**Figure 5:**
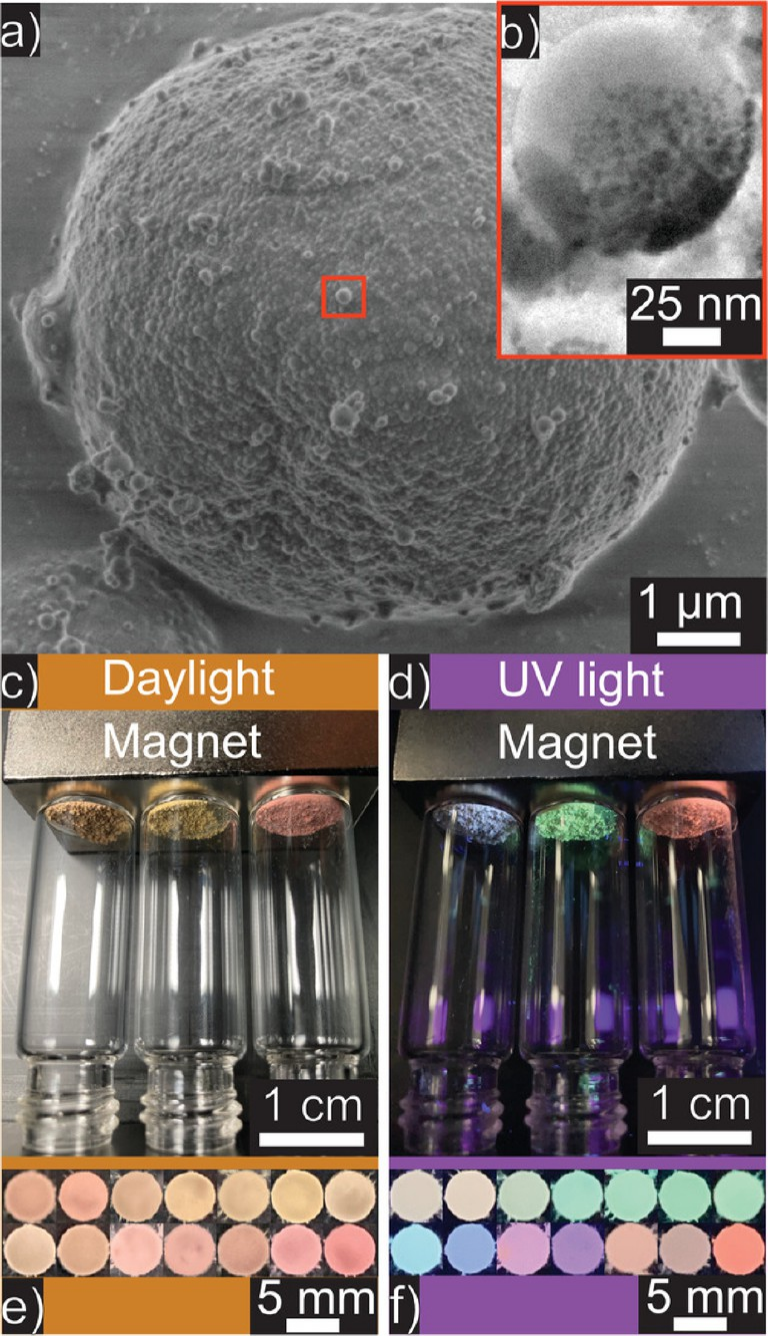
(a) SEM image of the magnetic fluorescent PS supraparticles with (b) a TEM image of the constituent iron oxide nanoparticles. The colored fluorescent emission of each dyed supraparticles is visible when viewing in (c) daylight versus (d) UV light. (e,f) Color variations are also shown using different supraparticle blends. Reproduced from ref (34) under the Creative Commons CC BY license. Copyright 2022 The Authors.

The authors investigate the use of three UV fluorescent organic molecules as the basis for their luminescent identification: two coumarin derivatives (Figure 3f,g) and a rhodamine species.^34^ These were used to cover the blue, green, and red regions of visible light emission, though the authors also proposed three additional organic dyes that could also be used to further exploit the visible spectrum range. Dyes were encapsulated in PS nanoparticles (180 nm average diameter) using an emulsion solvent evaporation method, with dye loadings between 0.010 and 0.725 wt %. These fluorescent nanoparticles were then combined with similar magnetically identifiable nanoparticles and spray-dried to form encoded micron-scale supraparticles (see Figure 5). This hierarchical design allows for the formation of a huge variety of coded particles, though omission of the magnetic component will likely still yield enough unique markers for the purpose of identifying different plastic waste streams.

This particular methodology is unlikely to be suitable for producing the very large quantity of PS used in, for example, the packaging industry. However, a small quantity of these fluorescent nanoparticles could easily be added to PS masterbatches in the extrusion process to label them. As such, this emulsion-solvent evaporation method could be used as an alternative to produce organic fluorescent markers that are highly compatible with commodity plastics instead of chemically modifying the dye structure with long aliphatic moieties, as seen in previous examples.

Various patents also detail the use of organic fluorescent molecules in tagging polymeric articles for selective identification.^26,47−50^ Varying degrees of technical detail are provided with most claiming a broad scope for implementation of the inventions in identifying or authenticating polymer products. In a notable example by Hubbard et al., an invention is described using any organic, inorganic, or organometallic compound whose excitation wavelength is between 100 and 1100 nm, with emission wavelength <250 nm and temperature stability of above 350 °C.^35^ A total of 91 possible commercially available dyes is given, including many in the classes of UV organic fluorophores already mentioned in this section. Concentrations of these compounds are said to vary between around 10^–12^ to 0.05 wt % of the polymer substate, dependent on the quantum efficiency of the selected dye.

The authors detail an example whereby Lumogen Red 300 (another perylene derivative) was incorporated into the melt polymerization of polycarbonate and subsequently heat treated at 400 °C.^35^ No significant reduction in the fluorescent emission of the organic dye was observed in the final article, thereby indicating this organic dye would be suitable for labeling polycarbonate products such as data storage disks. Though polycarbonate products do not necessarily make up a large portion of postconsumer plastic waste, the invention is useful for demonstrating how the organic fluorescent markers could be applied to plastic feedstocks in the early stage of polymerization rather than compounding into existing resins.

#### 3.1.2 Inorganic Photoluminescent Markers

In addition to the organic UV fluorescent molecules already discussed, a host of inorganic UV photoluminescent compounds, most prominently containing lanthanides, can also be applied to the labeling of plastics. Broadly speaking, the photoluminescence quantum yield of most inorganic dyes is far less than their organic counterparts. However, such compounds are generally more chemically stable and, hence, their detection lifetimes can theoretically be much longer than many organic dyes.^41^

In an example from Massardier et al., three inorganic UV photoluminescent compounds were blended into clear and black PP during extrusion at a concentration of 1000 ppm.^29^ The commercially available compounds selected were an aluminum barium magnesium oxide, a doped aluminum and barium oxide, and a doped vanadium trioxide. Each proved to emit at wavelengths of around 520, 450, and 620 nm respectively, when irradiated with UV light (325 nm). The photoluminescent emissions of the tracers were found to be detectable in all PP composites, although, in the black PP, the photoluminescence intensity was shown to be significantly reduced, thereby potentially limiting its detectability if a smaller quantity of inorganic dye were demanded.

The authors found that a homogeneous dispersion of the inorganic dyes within the PP matrices could be achieved when applying high shear rates in the extrusion process (see Section 4.1), with inorganic particulates being around 80 nm in size. However, inclusion of the tracers was found to slightly increase the crystallinity of the PP polymer and led to a small increase in the Young’s modulus (+20 MPa) and impact strength (+6 kJ/m^2^). The change in mechanical properties is certainly too small to make any notable difference in simple applications such as packaging. However, it does pose a potential issue if the method were to be applied to high-performance polymers, for example.

Another potential advantage of inorganic dyes when compared with organic ones is, broadly speaking, the increase in photoluminescence lifetime. Some inorganic compounds can emit visible light several milliseconds, or longer, after excitation. This is due to the fact that these compounds instead express phosphorescence, a different radiative process whereby emission continues long after excitation has occurred and can be several orders of magnitude longer than the emission lifetime of organic fluorescent dyes.^40^ This feature could prove advantageous in plastic sorting when wanting to improve the reliability of detection. In principle, stray fluorescent signals from organic contaminants or additives can be filtered out of detection since their emissions decay much faster.

**Figure 6:**
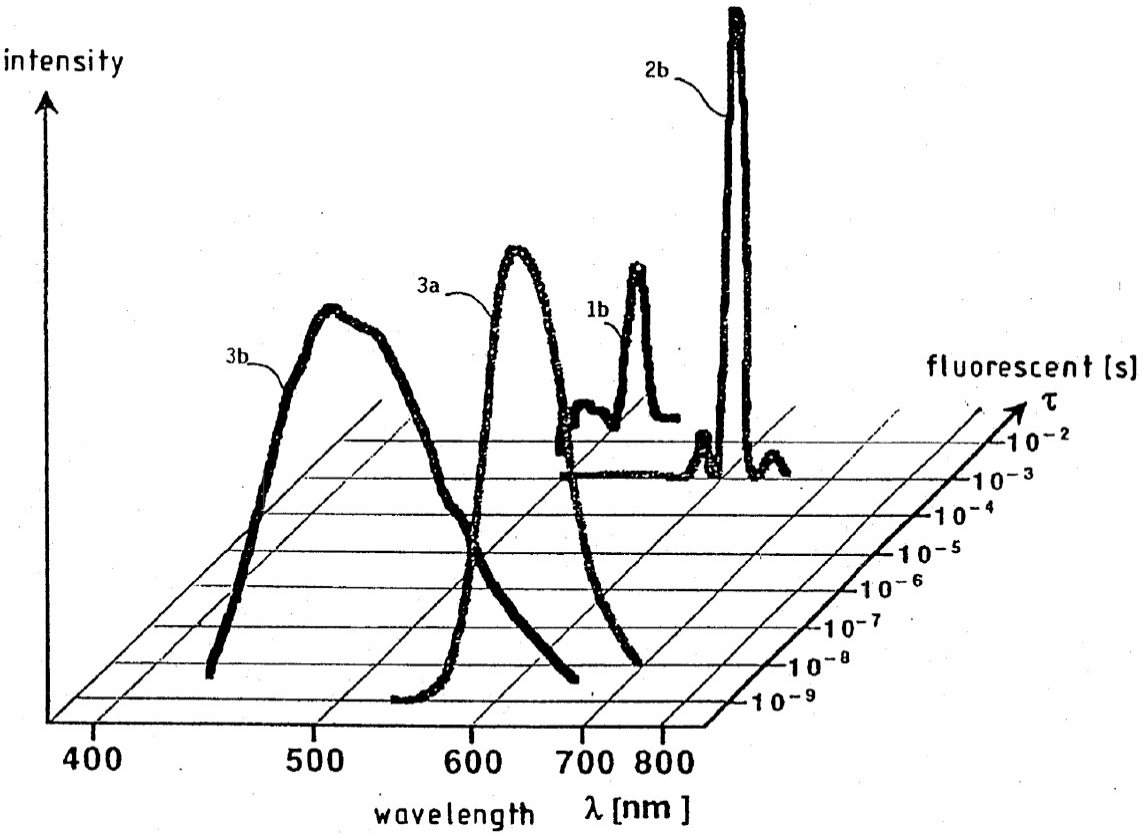
Two-dimensional photoluminescent emission spectra of LDPE labeled with the terbium complex (1b), erbium complex (2b), and two coumarin dyes (3a,b). Reproduced from ref (30). Copyright Bayer AG.

A patent filed by Becker et al. details the use of a combination of organic and inorganic photoluminescent dyes to identify plastic articles by means of both their wavelength of emission and photoluminescence lifetime.^30^ The invention gives specific details on the synthesis of two polymeric inorganic photoluminescent compounds. Both dyes are formed of cross-linked poly(stearyl methacrylate) containing small quantities of either a terbium or europium complex with polymerizable ligands. Further instructions for mixing the dyes into low-density polyethylene (LDPE) are also provided. The invention then demonstrates that the two labeled LDPE samples can be differentiated by their characteristic emission wavelengths. They then go on to show that these can also be distinguished from two coumarin organic fluorescent dyes loaded into the same LDPE sample by recording emission after an 80 ms pause upon the termination of excitation source. This is notwithstanding the fact that the emission wavelengths of the different species of dye overlap in the visible spectrum (Figure 6). Therefore, it would be possible to use a mixture of organic and inorganic labels to widen the possible binary encoding of different plastic articles, even if photoluminescent emissions of components overlap. However, the success in detecting these different lifetimes in a large-scale industry setting has yet to be demonstrated.

Another more recent patent developed by Harris et al. also uses inorganic compounds to sort waste polymeric items on the basis of their optical photoluminescence emission.^51^ Similarly, this patent also utilizes the slow emission decay of certain inorganic phosphors for efficient detection, such as the long-persistent phosphor Y_2_O_2_S:Eu^3+^.^52^ However, this invention improves upon the previous by recognizing that the detection of inorganic dyes with phosphorescence of even several milliseconds can prove troublesome in practice. When attempts are made to detect these dyes a few milliseconds upon the end of excitation, the emission intensity is significantly reduced, thereby necessitating the use of higher quantities of dye to enable detection. The authors instead propose the use of certain inorganic phosphors, such as Y_2_O_2_S:Eu^3+^, that can be stimulated to release stored energy upon exposure to a secondary light source in the infrared region. This method not only improves the reliability of the detection of inorganic photoluminescent labels at low loading but also lends itself well to integration in the current waste management industry because near-infrared (NIR) light sources are already heavily employed.

In one of the most recent publications on the use of photoluminescent markers in commodity plastics, Yin et al. took a slightly different approach by incorporating SiO_2_ nanoparticles into a PE solution.^36^ The SiO_2_ nanoparticles in this study were not designed to be emissive and are instead used in the method as an active site to partially degrade PE under moderate heating into photoluminescent carbon quantum dots (CQDs). CQDs are a small particulate (<10 nm) allotrope of carbon, often with some type of surface passivation.^53^ These newly formed CQDs showed a clear radiative emission maximum between 394 and 408 nm, depending on the preparation conditions. This was achieved by heating the PE/SiO_2_ nanoparticle mixture in toluene for 24 h between 90 and 110 °C, with the increasing reaction temperature used to reduce the emission wavelength of the CQDs.

**Figure 7:**
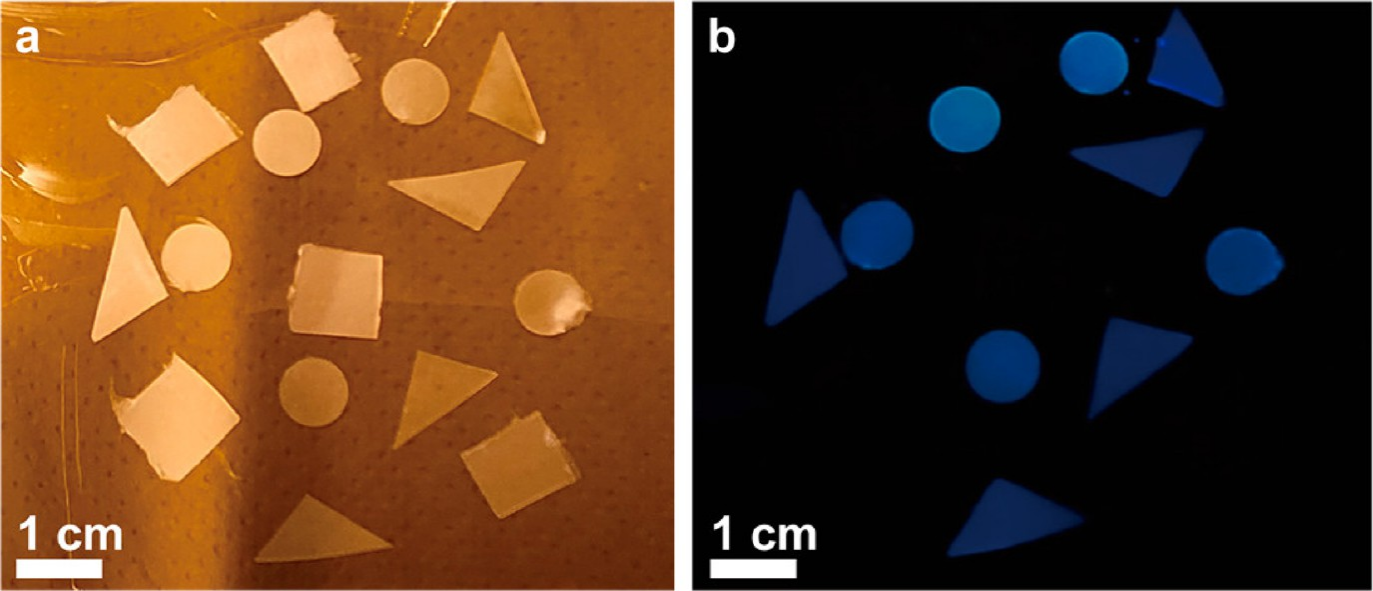
Digital photographs of PE samples under (a) daylight and under (b) a 367 nm light source. The sample shapes relate to pure PE (rectangles), the crude PE material containing CQDs and SiO_2_ (circles), and PE containing only the isolated CQDs (triangles). Reproduced from ref (36). Copyright 2021 American Chemical Society.

The synthesized CQDs could be extracted from the residual materials and reinserted into virgin PE to label them, or alternately, the crude reaction material could be mixed directly with virgin PE at a 10 wt % loading.^36^ Both forms of CQD-labeled PE were visibly distinct from unlabeled PE when viewed under UV (367 nm) light (Figure 7). However, it is not yet clear whether the additional incorporation of the degraded PE and SiO_2_ nanoparticles has an appreciable effect on the mechanical properties of the labeled PE. A 10 wt % loading of this additional material may be significant enough to downgrade the properties of a PE resin, such that it is difficult to process using current industrial methods.

Elsewhere in the literature, there are several more articles that detail the use of UV-excitable photoluminescent markers to label plastic articles.^54−57^ Excitation by UV or high-energy visible light has consistently been the most popular mode of photoluminescence proposed for the labeling of commodity polymeric materials. However, in many cases, these publications fail to mention the details of the specific chemical dyes utilized and instead focus on the specifics of the detection and sorting processes employed.

Though UV excitable photoluminescent markers boast very large flexibility in terms of chemical structures, and their detection is rather trivial, there are some downsides to their use in waste sorting. A key disadvantage is that their detection relies on introducing new UV excitation light sources into existing waste management infrastructure. This not only represents a large capital cost but can also be a safety hazard if not implemented correctly. Any reflective contaminants in the waste stream have the potential to redirect the light source toward facility personnel and expose them to the harmful effects of UV radiation.

### 3.2 Infrared Photoluminescent Markers

A solution to the pitfalls of photoluminescent markers requiring excitation by UV–vis radiation can be to utilize already existing NIR light sources as the excitation source for photoluminescent detection. During conventional photoluminescent, this infrared radiation would be re-emitted as a lower-energy photon that would be impractical to detect. However, some chemical compounds can undergo a photoluminescent up-conversion (UC) or anti-Stokes fluorescence, whereby the emitted radiation is of higher energy than that absorbed.^58^ This is achieved by a two-photon absorption process through an intermediate excited state, with stored energy emitted as a single combined photon in the visible light region.

**Figure 8:**
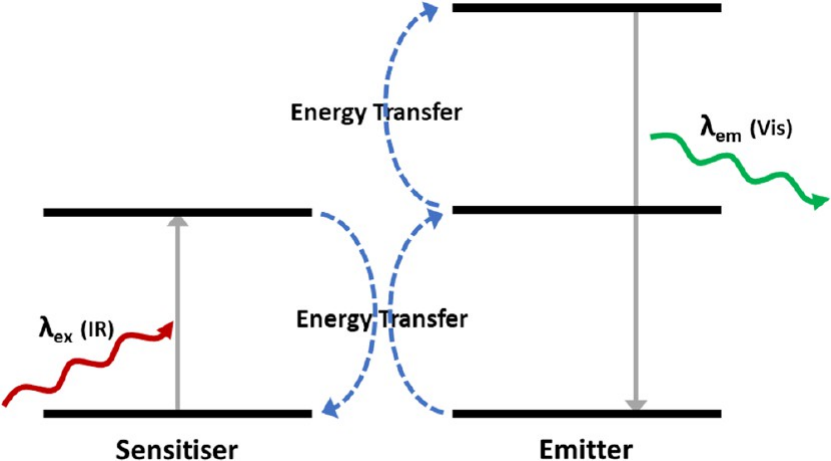
Schematic demonstrating the mechanism for the photoluminescent up-conversion process.

This phenomenon was originally demonstrated by Auzel in 1966 using a Yb^3+^ material, and since then, the range of UC-capable materials has expanded greatly.^59^ Though some organic materials have been reported to undergo this process, most materials studied are inorganic crystals.^60^ There are several possible mechanisms to combine the energies of two photons, which often involve the use of a sensitizer component (e.g., Yb^3+^) to absorb low energy photons before transferring multiple doses of this energy to an emitter component that releases the combined energy (Figure 8). Further information on this process can be found elsewhere.^58,61^

**Figure 9:**
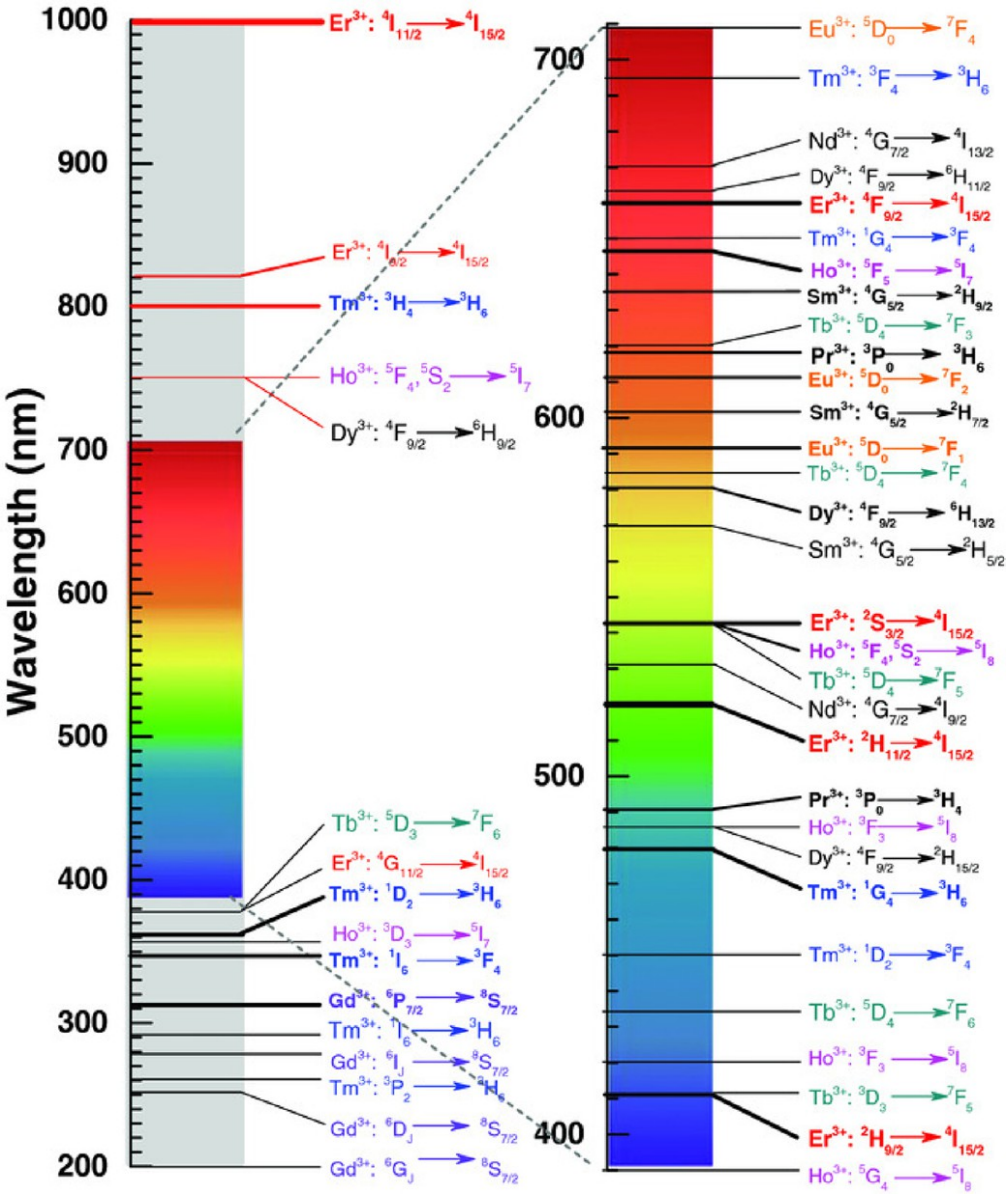
Main UC electronic transitions for Ln^3+^ ions, shown on the UV–vis–IR spectrum, to indicate the energy of the emitted UC photon. Reproduced with permission from ref (62). Copyright 2017 Wiley.

A review paper by Gao et al. discusses the optical properties of trivalent lanthanide (Ln^3+^)-doped inorganic UC materials and their relevance for use in photoluminescent labeling of plastic waste.^62^ The authors highlight some of the key advantages of UC photoluminescent labels over UV photoluminescent alternatives, with the main point being that there is a very high signal-to-noise ratio because of the lack of any stray fluorescent signals from UV–vis excited contaminants. They also show that because of the extensive range of electronic transitions available to Ln^3+^ ions, the emission wavelength can be tailored to a vast array of values in the NIR-to-UV range on the basis of the chosen ion (see Figure 9). This, combined with the very sharp emission peaks of Ln^3+^ UC materials, offers many possibilities for the spectral coding of plastic materials by combining luminophores.

**Figure 10:**
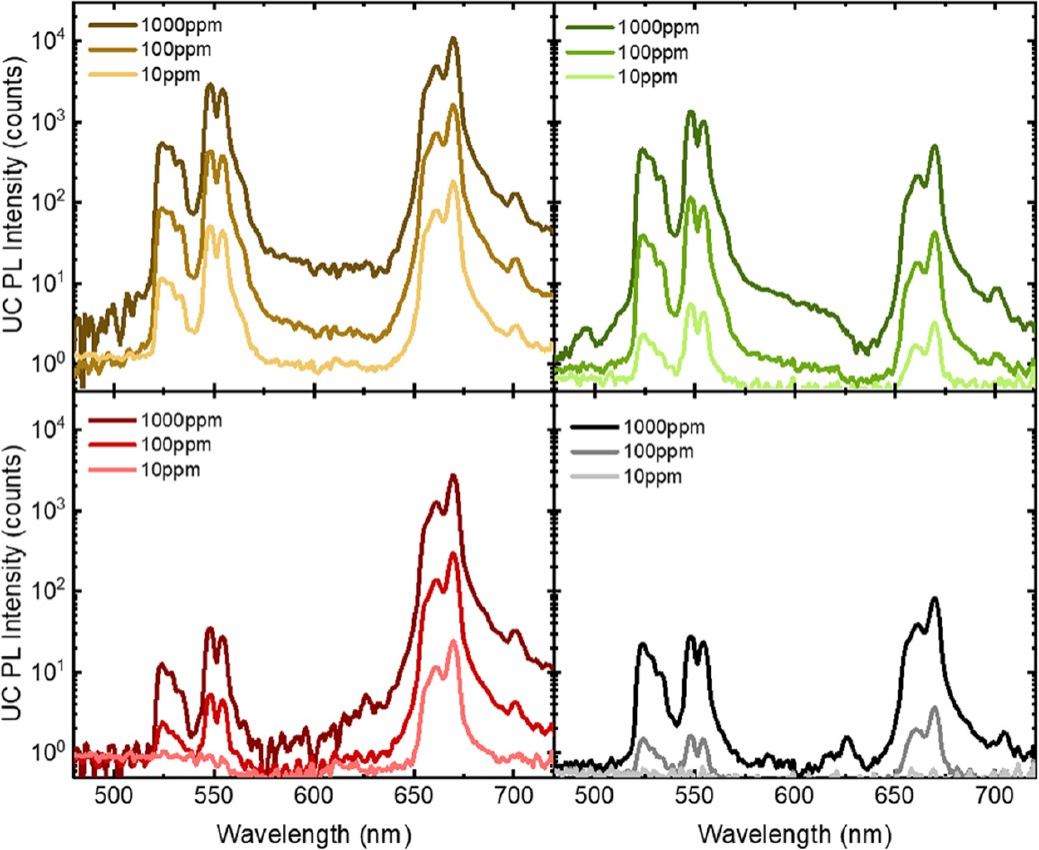
Up-conversion photoluminescent (UC PL) spectra of different semitransparent colored HDPE labeled with various quantities of the Yb^3+^/Er^3+^-based marker. The HDPE colors were (top left) yellow, (top right) green, (bottom left) red, and (bottom right) black. Light source: λ_ex_ = 980, 10 W/cm^2^. Reproduced with permission from ref (31). Copyright 2020 Elsevier.

A study by Woidasky et al. tests the feasibility of using UC photoluminescent materials in colored HDPE.^31^ A UC luminophore comprising a Yb^3+^ sensitizer and a Er^3+^ emitter component was compounded into partially transparent yellow, green, red, and black HDPE films. Upon excitation with a 980 nm light source, the characteristic emission of the UC luminophore (λ_em_ = 540 and 670 nm) could be detected in all colored films at a dye loading of at least 100 ppm (Figure 10). However, this detected emission was significantly reduced for the black and red colored films. For the red film, this was due to the overlap between the emission of the dye and optical absorbance of the colored pigment in the HDPE. For the black film, the authors note that the very low emission signal is a result of both the absorption of the emitted light and strong absorption of the IR excitation source. This issue resonates with the existing well-known problems in sorting black plastics articles using NIR sources, whereby the black pigments absorb all light in the visible and IR regions.^24^

The authors also incorporated the UC dye into several colored HDPE bottles that were fully opaque.^31^ The labeled HDPE was extruded and then blow molded into a white, green, and black bottle. Characteristic UC emission for the dye could be detected at a concentration as low as 10 ppm for the white bottle and 100 ppm for the green bottle. However, even at loadings as high as 1000 ppm, no optical emission was detected for the black-colored bottle. The lack of photoluminescent intensity was ascribed to the same reasons outlined for the thin HDPE films, which highlights the potential incompatibility of UC fluorescent markers with black or dark colored plastics. Nevertheless, some black pigments that do not absorb over all IR wavelengths have become available in recent years and will likely be more compatible with this type of photoluminescent compound.

Though UC photoluminescent markers offer several advantages over other photoluminescent labeling options, there is a significant disadvantage to using these materials. As a nonlinear luminescent process, the emission intensity of UC photoluminescence is generally much lower than conventional photoluminescent mechanisms, with quantum efficiency limited to a theoretical maximum of 50%.^63^ Furthermore, in order to induce the quick sequential absorption of multiple photons, a high-intensity photon pump source may also be required to achieve a detectable emission intensity, such as a laser. This counteracts one of the main advantages of using an NIR excitation source, in that new, more powerful excitation sources may need to be installed in sorting facilities and would carry health hazards if not properly contained.

### 3.3 X-ray Fluorescent Markers

In addition to the other excitation sources discussed, characteristic photoluminescent emission can also be induced by using an X-ray source. X-ray fluorescence (XRF) can be used to identify specific elements in a sample, including even their oxidation states. Irradiation of a sample with high-energy X-rays can remove electrons from the inner orbitals of its atoms. Outer electrons then “fall” to fill these holes and, in the process, emit a photon of characteristic energy related to the electronic structure of that element. The process is often used in elemental analysis.^64^

XRF has previously been applied to the sorting of plastic waste. At present, it is limited to the removal of PVC from waste streams, particularly to separate from PET, which can be challenging because of their similarity in density.^65^ It is vital to remove PVC from the waste stream to prevent the accidental formation of hazardous chlorinated compounds upon reprocessing of the PET recyclate. Kenny and Bruner reported the use of XRF detection to separate PVC at a large sorting facility in 1992 and achieved a 99.5% rejection rate of PVC bottles at a capacity of 2270 kg per hour.^66^ Other work also investigates the viability of XRF for the removal of plastics containing hazardous brominated flame retardants from the waste stream.^67^

Though the results are impressive for sorting PVC, XRF cannot be used to separate other commodity plastics as these are all hydrocarbon-based with no distinguishing elements. This limitation can be overcome by inserting a chemical compound into a plastic resin with a unique identifiable element. This differs from the previous photoluminescence methods discussed, as detection of the compound relies only on the chemical marker having a unique element in its makeup, rather than depending on the whole compound’s specific interaction with a light source.

In a publication by Bezati et al., a detailed selection process is outlined to identify a promising collection of compounds suitable for XRF sorting of plastic waste.^37^ The entire pool of elements from the periodic table were gradually narrowed down on the basis of key criteria that would limit their use in plastic sorting, thereby leaving several suitable candidates. Many elements were easily eliminated on the basis of their physical state (e.g., noble gases), low XRF yields (elements with >30 atomic number), high toxicity (e.g., lead, arsenic) and high radioactivity (e.g., actinides). Further elements were eliminated on the basis of their high cost, such as the noble metals group. Additionally, common chemical compounds used in polymer production were considered, such as lubricants, fillers, colorants, stabilizers, antifoaming agents, and flame retardants. Common elements used in these applications were also omitted to reduce the likelihood of mischaracterization of labeled plastics. From this, it was concluded that rare earth metal oxides provided the best potential for use as XRF tracers in plastics. Considering the production quantities and reserves of several of these, as well as the potential hazards for these to be incinerated rather than recycled, Y_2_O_3_, CeO_2_, Nd_2_O_3_, Gd_2_O_3_, Dy_2_O_3_, Er_2_O_3_, and Yb_2_O_3_ were concluded to be the most suitable candidates.

**Figure 11:**
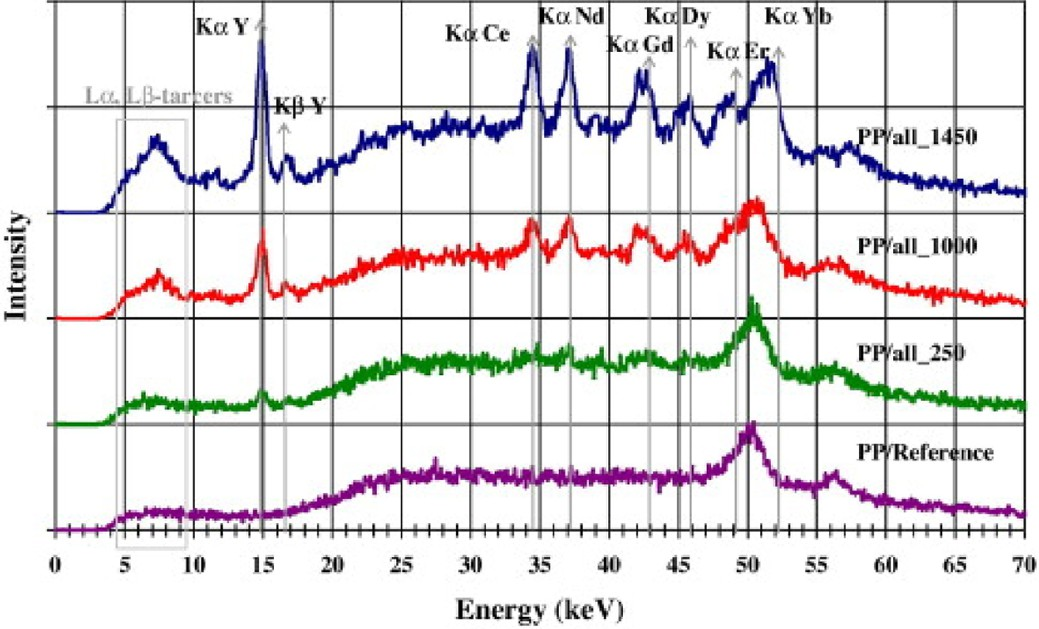
XRF spectra for PP samples mixed with all seven rare earth metal oxides at concentrations of 0.145 wt % (blue), 0.1 wt % (red), 0.025 wt % (green) and 0 wt % (purple). Reproduced with permission from ref (32). Copyright 2010 Elsevier.

Subsequently, Bezati et al. tested these metal oxides as XRF tracers by blending them into PP.^32,39^ When all seven metal oxides were simultaneously mixed into PP at a concentration of 1000 ppm each, five of the tracers were clearly visible by their *K*_α_ emission lines in the fluorescence spectrum (Figure 11). Er_2_O_3_ and Yb_2_O_3_ could not be distinguished because of their overlap with a strong tungsten emission from the X-ray source. It was found that even when loading Nd_2_O_3_ and Gd_2_O_3_ into PP at concentrations as high as 1 wt %, the metal oxide fillers were dispersed homogeneously and had no noticeable effect on the melting temperature or crystallinity of the PP. However, in a similar result to Massardier et al.,^29^ a small increase in the Young’s modulus (+10–20 MPa) and reduction in the elongation at break (−10 to 15%) was noticed in the labeled PP. This minor increase in brittleness from neat PP is unlikely to pose significant issue for the vast majority of applications.

As the authors point out in their work, this method of tracer identification is especially useful for black or darkly colored plastics, such as those used in the automotive industry, because X-ray detection is not impeded by these colorants in the same way UV–vis and NIR sources can be. However, in these studies, tracer detection was only possible with long X-ray exposure times (1–4 min) to detect the 1000 ppm of metal oxides. This acquisition time needs to be reduced to as little as 10 ms to match current high-speed waste sorting lines.^68^ Bezati et al. point out that much more intense X-ray sources are commercially available than their apparatus to help with this issue. However, as with the use of UV light sources, the cost and safety implications of using these high-dose X-ray sources must be considered.

### 3.4 Time-Resolved Photoluminescence

As previously mentioned, photoluminescence can be characterized not just by the excitation and emission wavelengths, but also by the emission lifetime. This is the time a compound will remain excited before emitting the absorbed energy. When an excitation source is removed from a photoluminescent material, the intensity of the emission will decay exponentially. The rate of this decay determines the emission lifetime, which may subsequently be used as a way of identifying the compound.^40^

As previously discussed, Becker et al.^30^ took advantage of the large difference in photoluminescence lifetime between organic and inorganic markers as a means of identification. However, the inherent autofluorescence of some polymers can also be used as a means of identification, without the inclusion of any specific markers. Langhals et al. reported the identification of several high-performance technical blend polymers, including Luran (polyacrylonitrile–styrene copolymer), Delrin (POM), and Ultramid (polyamide with glass fiber).^69^ Though the autofluorescence of these polymers would be very difficult to differentiate solely from their similar broad emission profiles, their emission lifetimes (τ) differ by a few nanoseconds and can be used as an alternate means of detection. They also showed that this method of photoluminescent detection can accommodate the inclusion of several perylene fluorescent dyes (Figure 3e). Inclusion of the markers leads to further small variations in τ that may be used as a means to identify special batches of the technical polymers.

**Figure 12:**
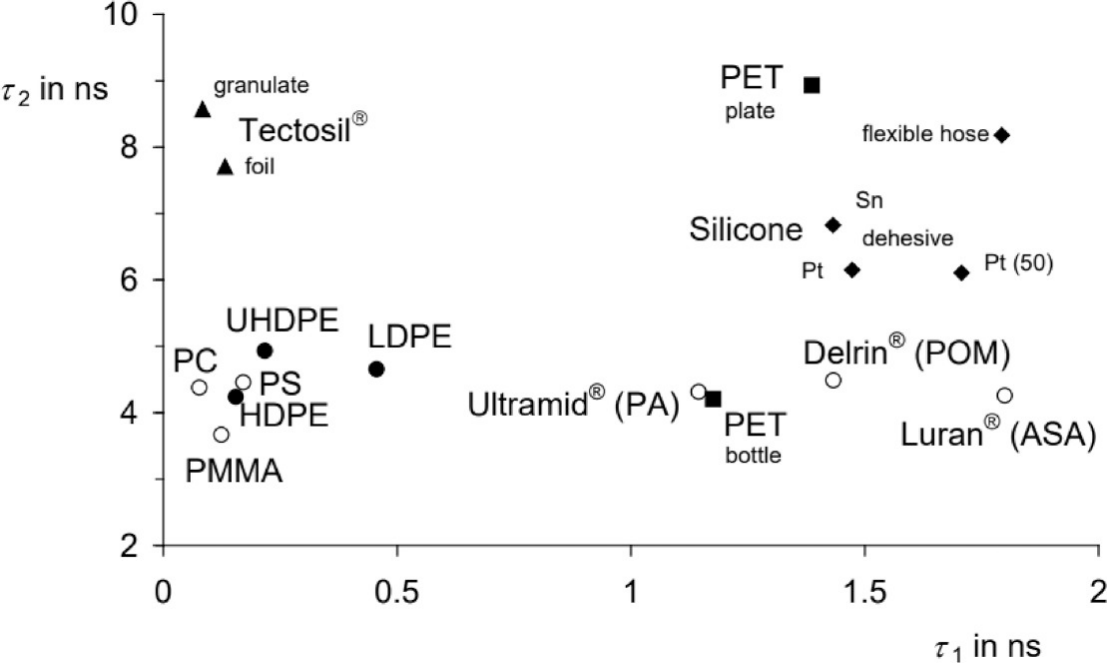
Two-dimensional characterization of various polymers on the basis of their biexponential fluorescent decay constants, τ_1_ and τ_2_. Symbols relate to different types of PE (filled circles), PET (squares), silicone dehesives (diamonds), the silicone elastomer Tectosil (triangles), and other commodity and technical polymers (unfilled circles). Reproduced from ref (38) under the Creative Commons CC BY license. Copyright 2015 The Authors.

A later publication, again by Langhals et al., improves the performance of this photoluminescent lifetime detection.^38^ Here, the emission lifetime, τ, is measured as a biexponential decay model, which gives two distinct time constants (τ_1_, τ_2_) instead of one. This two-dimensional characterization allows for greater resolution between autofluorescent polymers that may have similar single exponential decay constants (Figure 12). Thus, a variety of technical grade and commodity polymers can be recognized. Furthermore, the technique was also shown to be capable of distinguishing LDPE from HDPE and PET molded into either bottles or plates, possibly because of small differences in their crystallinity. PET exposed to lipophilic contaminants (diesel and engine oil) could also be identified separately from neat PET because of the oils’ slight plasticizing effect on the polymer.

Overall, the method of identifying plastics on the basis of only the emission lifetime of the polymeric material shows great promise. The key advantage is the high selectivity between autofluorescent polymers without the need for any markers to be applied. The methodology has also been particularly useful for developing new techniques for the characterization of microplastic wastes.^70^ However, the method has yet to be exploited in larger scale waste sorting facilities. This may be because of the high precision needed to accurately determine the fluorescent lifetimes (<0.1 ns), which could lower the sorting success rate. Additionally, the observed change in emission lifetimes between polymers exposed to different materials does pose a question as to how robust the method would be for typical postconsumer nonpristine plastic waste streams.

## 4 Methods of Incorporating Photoluminescent Labels

Another key consideration when seeking to use photoluminescent markers to label polymeric items is how the dyes will be incorporated into the product. As mentioned briefly in the previous section, a few different approaches can be taken. A summary of the advantages and disadvantages of each approach is shown in Table 3. These are discussed in more detail below in Sections 4.1, 4.2, and 4.3.

**Table 3:** Assessment of Different Strategies to Incorporate Photoluminescent Markers into Polymers

	polymer extrusion	surface coatings	external labels
visibility	medium	high	high
How easily can the marker be detected?	Dependent on how homogeneous the marker is and the transparency of the polymer.	Marker is in high concentration on polymer surface.	Marker is placed externally above the polymer material.
compatibility	medium	low	high
How easily can markers be incorporated?	Extrusion must be optimized to ensure good dispersion of marker in the polymer.	Marker must be formulated into a suitable surface coating. Marker may also need to be food contact safe.	Marker need only be compatible with the external label rather than the polymer.
separability	low	medium	high
How easy is it to separate the markers from the polymer?	Once marker is extruded into polymer it is almost impossible to remove.	Surface coating may be washed off during recycling. This must be optimized for each formulation.	Removal of labels is a step already required in many recycling procedures.
reliability	high	medium	medium
How likely is it that the marker will remain detectable?	Polymer can be identified even in granulated form.	Surface coatings could be partially rubbed/washed off prior to sorting.	Label could fall off prior to sorting; cannot be used for granulated material.
viability	high	low	medium
How easily can the method be adopted by industry?	Markers can easily be added to masterbatches of polymers.	New formulations must be made to suit marker, polymer, and application. Applying coating may introduce a new manufacturing step.	Addition of markers to the print process for external labels may be relatively straightforward.

### 4.1 Polymer Extrusion

The most common method applied in the literature to include photoluminescent markers into a commodity plastic is by polymer extrusion. A granulated polymer feedstock is fed into a heated screw barrel, along with the photoluminescent compound(s) and any other additives. The markers then mix with the molten polymer before being pressed through a die to shape the resin and yield a continuous flow of the labeled polymer.^71^ In this way, the photoluminescent molecules used to label the plastic are directly incorporated into the polymer matrix. Polymer extrusion is already used extensively in industry, which makes the large-scale adoption of this method of labeling relatively trivial.^72^

This method proves to be widely applicable to all the different types of photoluminescent markers already discussed, with working examples shown for UV–vis,^28,29^ IR,^31^ and X-ray-activated^32,39^ dyes. Only twin-screw extruder models were used in all these studies. The high shear and superior mixing of the twin-screw design over a single-screw geometry is likely required to achieve a homogeneous dispersion of the photoluminescent markers throughout the polymer.^71^ These reports in the literature have focused only on the use of HDPE and PP resins, as they are the two most widely produced commodity polymers.^1^ However, it is justifiable to believe that the coextrusion of photoluminescent markers is possible with a variety of other thermoplastics, as well, given that extrusion is a widely applicable processing method.

**Figure 13:**
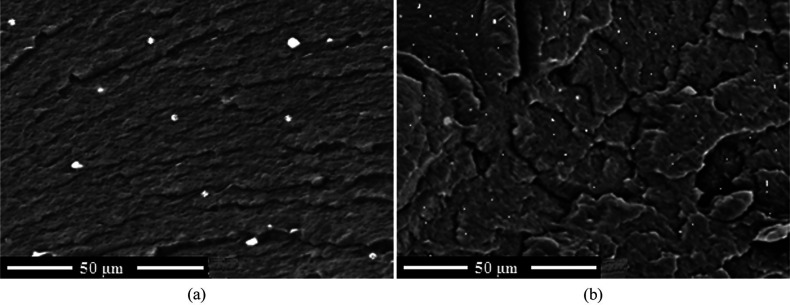
SEM micrographs of PP extruded with an inorganic photoluminescent marker at screw speeds of (a) 100 rpm and (b) 800 rpm. Reproduced from ref (29) under the Creative Commons CC BY license. Copyright 2015 ABPol.

Despite the regular use of polymer extrusion to incorporate photoluminescent markers into commodity plastics, almost all reports in the literature neglect to report how the markers are dispersed in the extrusion product. An exception to this is the publication by Massardier et al., where UV photoluminescent inorganic compounds are extruded into PP under different screw rotation speeds.^29^ A 0.1 wt % loading of marker was added to PP with extrusion rotation speeds varied from 100 to 1200 rpm. This equates to a shear rate of 140–750 s^–1^. The authors found that by increasing the rotation speed from 100 to 800 rpm, the size of the inorganic agglomerates seen in the PP was significantly reduced from around 2–5 μm down to less than 80 nm (Figure 13). Homogeneity in the sample was also greatly improved. This meant that when it came to measuring the photoluminescent signal of the marked PP samples, the sample processed at 800 rpm gave a lower standard deviation between measurements than the 100 rpm sample.

In addition, the authors also performed mechanical testing on the extruded PP materials.^29^ They discovered that, although any addition of the inorganic marker to the plastic caused some change in the mechanical properties, the lowest change was seen in the midrange screw speed. At the lowest speed (100 rpm) the PP was found to suffer from a reduced elongation at break (−50% compared with neat PP), attributed to the large inorganic agglomerations acting as fracture points. However, the largest difference in mechanical properties was seen at the higher screw speed (1200 rpm). A large reduction in tensile modulus (−170 MPa) and elongation at break (−120%) was observed compared with the PP reference. This was shown to be a result of degradation in the PP material due to the high shear stress during extrusion, with the temperature of the PP at the extruder exit increasing from 232 to 252 °C.

Overall, the studies suggest that as high a shear rate as possible should be applied during extrusion to maximize mixing, up to the point where the polymer begins to suffer high degradation due to self-heating. Though, it should be noted that these results only study the effect of adding crystalline inorganic photoluminescent markers to the extrusion process. This conclusion might not apply when considering organic or polymeric markers. There may also be some compatibility issues between thermally sensitive markers, particularly organic fluorophores, and certain thermoplastics. For example, PET is processed at around 280 °C because of its high melting point, hence, only markers stable above this temperature would be compatible in the extrusion process.^73^ It is also not yet clear if this method would be as successful when selecting markers with different physical forms, such as the supraparticles or CQDs previously discussed.

In general, polymer extrusion is a robust method of including various types of photoluminescent materials into a range of thermoplastics. Plastics physically embedded with these markers offer possibly the greatest reliability when it comes to the detection of a labeled plastic article. The labeled plastic can be significantly damaged or even fully granulated, and detection via the photoluminescent emission would still be possible when sorting. A loss of photoluminescence from leaching of the dyes into the environment may also be greatly reduced when compared with external coatings. However, this fixation of the markers into the polymer matrix can carry disadvantages, because once mixed, it becomes near impossible to remove them again. Separation of the markers during sorting may be valuable from an economic perspective, particularly in the case of rare earth metal oxides, and reduces the risk of mislabeling future recycled plastic feedstocks by accumulation of the markers.

### 4.2 Surface Coatings

**Figure 14:**
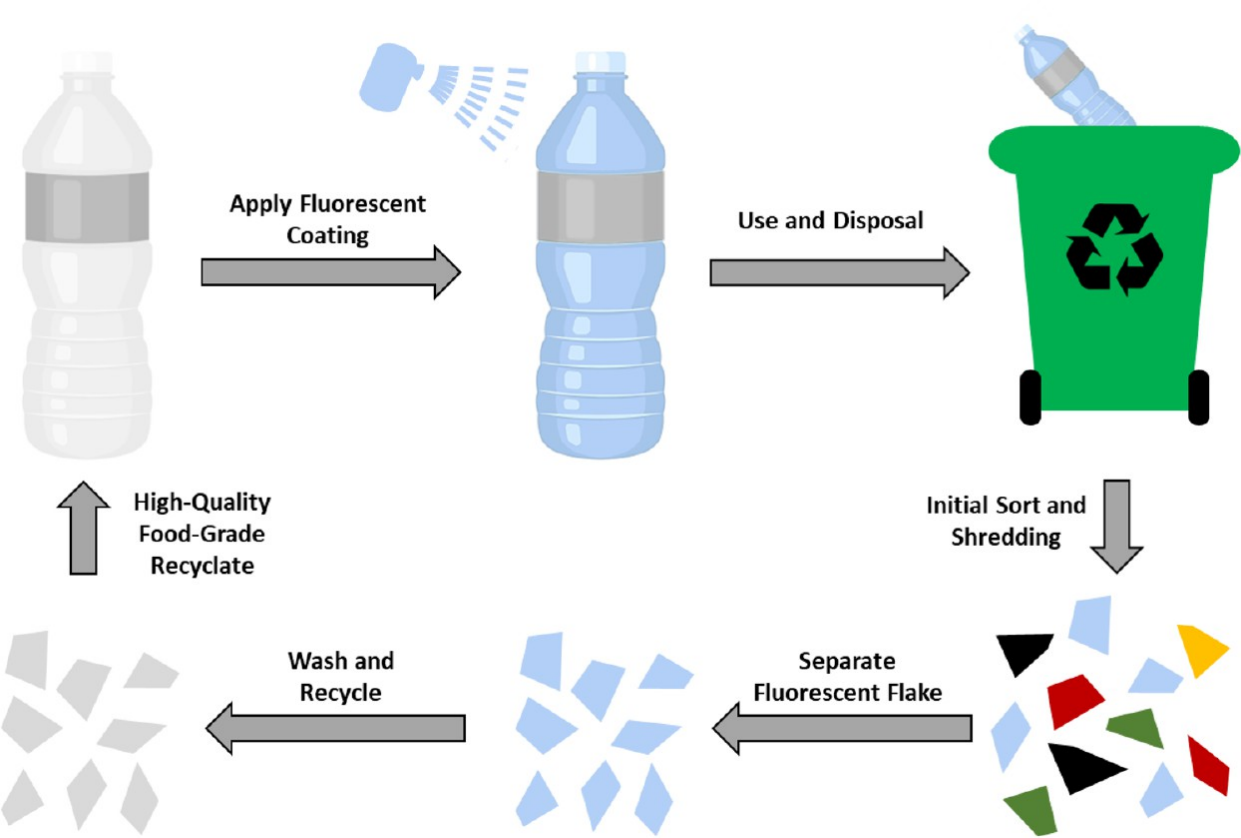
Schematic illustration of how surface coated bottles may be recycled.

A method to incorporate photoluminescent markers into plastic products which overcomes some of the limitations of extrusion blending is to coat the final plastic product with a surface layer of a photoluminescent dye (Figure 14). Thus, removal of the marker after sorting becomes much more achievable by applying a washing procedure, such as those already employed in the recycling process. A surface layer of photoluminescent material also allows for increased visibility of the dye.

The European Commission project, Polymark, explores this method of application on PET plastic bottles with the two organic dyes BBS and BBT, which are both food-contact-approved materials.^33^ The authors formulated water-based dispersions of each dye with the use of commercially available additives, which were then prepared into sprayable coatings. The dispersions were made from the dye, a stabilizer (Tween 80), defoamer (BKY012), and hyperdispersant (Solsperse 46000) under high shear mixing in water. The marker dispersions were then mixed into a styrene–acrylic copolymer (Neocryl A2092) along with a small amount of low-molecular-weight poly(ethylene glycol) (PEG_400_). The Neocryl was selected for its good compatibility with PET because of the similar functional groups of the styrene and acrylic combination, while the PEG_400_ was applied to aid in film formation.

The formulated spray coatings were applied in different concentrations to PET bottles. Even at the lowest concentration (0.1 wt %) the marker’s fluorescent signal could be distinguished at the bottle identified. Loading with a higher quantity of dye (1 wt %) resulted in a visually perceptible discoloration of the clear PET bottle, which is highly undesirable for the majority of end uses. They also found that detection of the photoluminescent coatings was much more challenging when applied to green colored PET bottles. Finally, washing procedures using a 2 wt % NaOH solution followed by either a hot water or caustic soda rinse were shown to efficiently remove most of the marker coating from coated PET flake.

Another notable observation from this study was the change in emission spectra of the BBS dye when either applied as the coating or compounded directly into the PET by extrusion. When extruded, the BBS exhibited a blue fluorescence (λ_max_ = 440, 470 nm), similar to that reported in literature when BBS is solvated in 1,1,1-trichloroethane.^74^ However, this emission shifts to a green color (λ_max_ = 505 nm) when applied as a coating, possibly because of the molecules’ more aggregated nature. This is an important factor to consider when applying any organic dye, as their emission properties tend to be particularly dependent on their surrounding environment.

Despite the success of this study, the incorporation of photoluminescent markers into coatings for plastic articles does face several drawbacks. The first is the added complexity in formulating the photoluminescent coatings. Though the method here could also be applied to other hydrophobic organic dyes, this would need to be reconsidered for the other types of markers discussed or when applying it to different polymer surfaces. The other limitation is the added safety restrictions on the photoluminescent marker, as high concentrations of the dye are expected to be in close contact with food or the user’s skin. This precludes the use of the vast majority of the photoluminescent markers discussed because of their potential toxicity, particularly those with inorganic content, thereby making the choice of dye extremely limited. The ecological impact of the release of these photoluminescent formulations into the environment during washing should also be considered.

### 4.3 External Labels

The final method to mark plastic objects with photoluminescent dyes is by their incorporation into external labels that can be attached to the article. This benefits from physically detaching the photoluminescent marker from the plastic it is used to identify, meaning a marker’s compatibility with a particular polymer need no longer be considered. Instead, the dye can be incorporated into an external material of choosing by, for example, either of the two methods already discussed. This label can then be affixed to the polymer article in question, where it must remain until the end of the plastic sorting step.

In the patent filed by Harris et al., a specific reference is made to the possible advantage of printing their persistent phosphors onto external shrink wrap labels that are present on a large number of plastic products, e.g. PET bottles.^51^ The external labels may be removed after sorting to prevent the photoluminescent materials from degrading the plastic recyclate, though this process is unlikely to be 100% efficient at large scale. Separation and collection of the marked labels also introduces the possibility of recovering and reusing the marker material.

**Figure 15:**
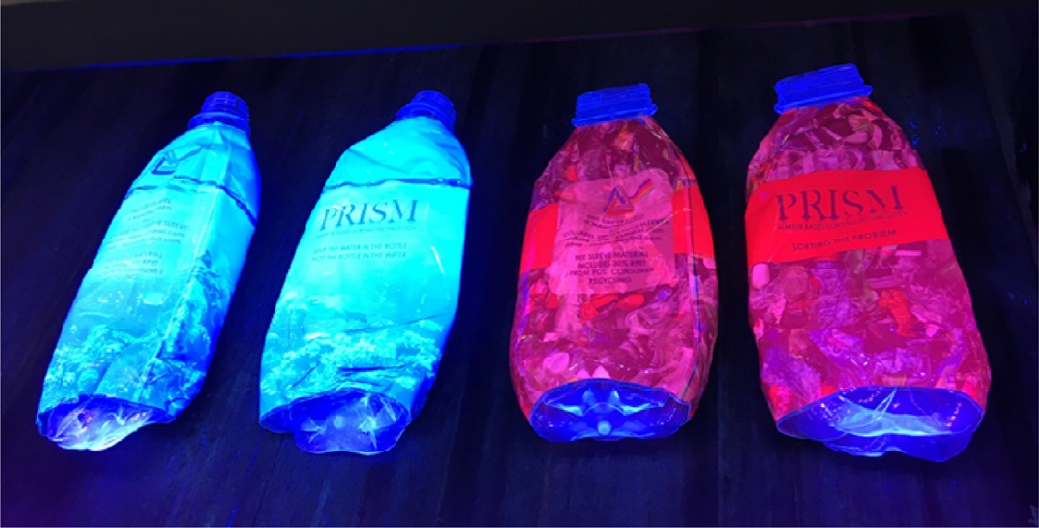
Plastic bottles labeled with Nextek’s PolyPRISM external labels viewed under UV light. Reproduced with permission from Nextek. Copyright 2020 Nextek Ltd.

This particular technology from Harris et al. was scaled up and piloted by Nextek under the name PolyPRISM.^75^ Here, as the authors describe, the phosphors are incorporated into the external bottle labels, which causes them to emit bright colors when exposed to the detector light source (Figure 15). Another potential benefit of this external wrapping is that the added label material can also serve to hide the polymer surface underneath. In the case of PET, for example, this blocks the visibility of its own autofluorescent emission that may otherwise obscure the detection of the marker.

The main disadvantage of using this approach is the greater likelihood of loss of marked plastic material due to removal of the external label before the sorting step. The label must be fitted securely to ensure it remains attached all the way from manufacture, through customer use and, importantly, during waste collection routes where the plastic objects are likely to be compressed and disfigured. This must also be finely balanced to ensure that labels can be removed on demand after sorting during the recycling process to prevent contamination of the recyclate. Moreover, the labels should themselves be capable of being effectively recycled, to avoid the generation of additional waste or contamination of waste streams.

## 5 Practical Modeling

### 5.1 Sorting System Design

A final key design consideration is how the photoluminescent-labeled plastics will be sorted in the waste stream. Efficient detection and separation of marked plastics is essential to ensure maximum purity of the recyclate, especially if this is intended for future food contact applications. Legislation on the purity requirements for recycled plastic intended for food contact are relatively open-ended because of the diverse nature of the problem, with both the FDA and European Commission currently opting to assess these standards on a case-by-case basis.^76,77^ However, scientific opinion published by the European Food Safety Authority (EFSA) currently recommends general limits for the maximum input of plastics from previous nonfood contact applications. This is to be no more than 5% for PET and no more than 1% for HDPE.^25,78^

**Figure 16:**
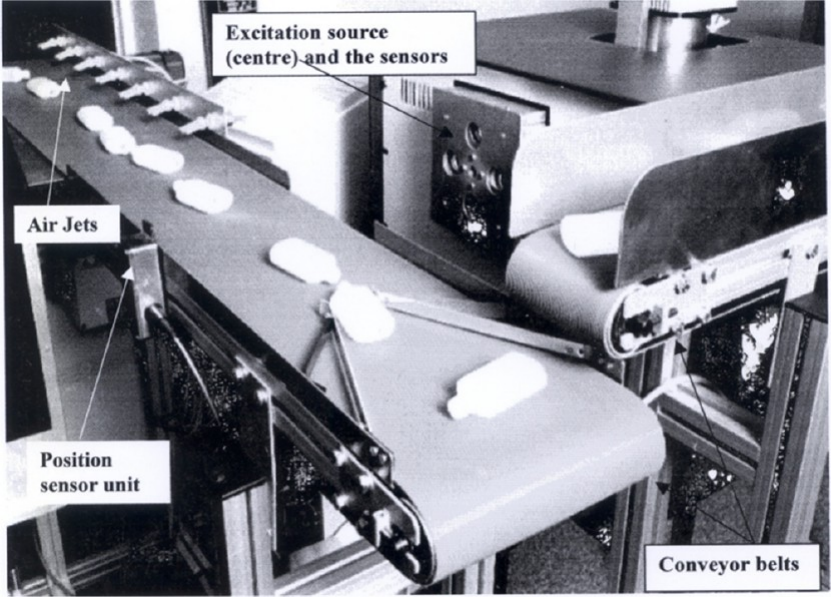
Annotated photograph of a laboratory scale apparatus for sorting photoluminescent-labeled bottles. Reproduced with permission from ref (55). Copyright 2004 Taylor & Francis.

Several publications demonstrate the use of small-scale sorting rigs to assess how efficiently their labeled plastics can be sorted. Earlier works on photoluminescent labeling by Ahmad detail the construction of a small sorting rig to separate plastic bottles embedded with a binary combination of several different photoluminescent tracers (Figure 16).^54,55^ A single combined module was used for both excitation and detection of the markers, which were placed horizontally across the direction of the sorting belt. The module comprised a central optical excitation source surrounded by four detector units. Bottles were physically separated on the conveyor belt using a positioned lateral air jet to deposit the bottle in the correct bin.

Fluorescent spectra were obtained as quickly as the electronic instrumentation would allow (around 4 kHz), with bottle categorization based on a voting protocol.^55^ In this way, the photoluminescent intensity of each spectrum counts as a vote toward a particular category. A high proportion of votes toward a particular category means it will be sorted, thereby reducing the chance of false identification. This lab-scale setup was then trialed in a larger scale industrial sorting plant, using a belt speed of 3.5 m/s. They found using their system that a maximum of 95% sorting purity could be achieved. However, the 5% impurities were determined to be mostly a result of mechanical issues in singulation of items on the conveyor belt and blow ejection irregularities rather than mischaracterization. It is unclear whether this purity may be impacted by sorting a mixture of plastic items rather than uniform bottles.

**Figure 17:**
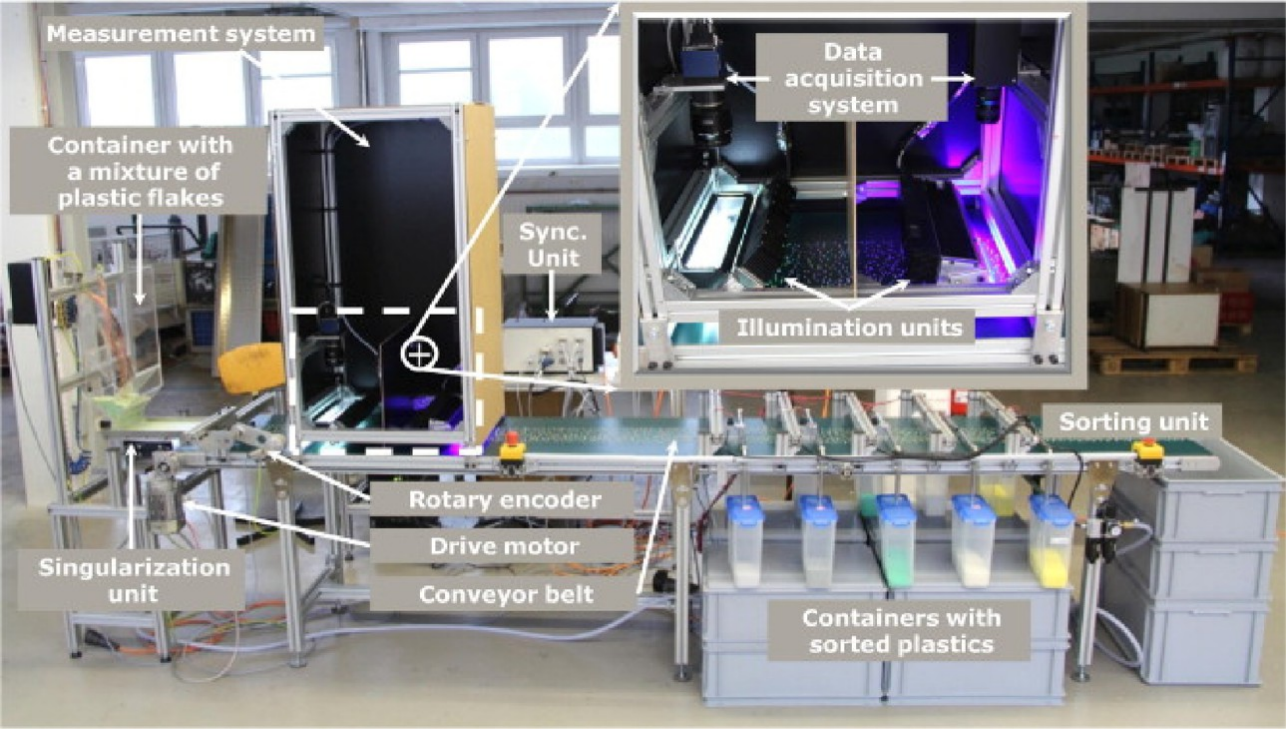
Annotated photograph of a laboratory-scale apparatus for sorting photoluminescent-labeled plastic flakes. Reproduced with permission from ref (57). Copyright 2015 Elsevier.

Brunner et al. reported the construction of another laboratory-scale sorting device, which provided excellent detail of their hardware and software concepts.^57^ The sorting unit contained many similar features to that of Ahmad’s design, with the main difference being its purpose to separate small plastic flakes rather than larger whole objects (see Figure 17). The system comprised a singularization unit to separate the flakes on the conveyor belt, as well as a scanning unit and sorting area where flakes were extracted into the correct collection bin. The scanning unit comprised two light sources and detectors. The first, a white light source, was used to track flake morphology, thereby allowing separation of all items. The second was a high-powered blue LED and spectral detector to induce and measure the photoluminescent emission of the markers. All components of the systems were linked by a synchronization module to control the feed to the singularization unit and belt speed.

The system’s performance was tested by separating feeds of three types of polymers [POM, polybutylene terephthalate (PBT), and acrylonitrile styrene acrylate (ASA)] divided into 12 different classes, each marked with a binary combination of up to four different photoluminescent markers.^57^ The 12th class of polymer contained no markers. Tests were conducted using a belt speed of 0.26 m/s to give an output of ∼250 kg of polymer per hour. All 11 marked flakes were separated with almost complete purity (98.8–100%). However, only a purity of 96% was obtained for the unmarked 12th class, with flakes of several of the photoluminescent-labeled polymers found as contaminants. This was deemed to be a result of inhomogeneous mixing of the markers in the polymers during extrusion, which resulted in some regions, and subsequently flakes, with very low emission intensities that were miscategorized as unlabeled polymer.

Beyond these laboratory-scale designs, recently Nextek has also reported success in industrial-scale sorting trials using a photoluminescent label marker (see Section 4.3) to sort food-grade PP.^79^ In their trial at a TOMRA test center in Germany, they reported achieving a sorting purity of 99.3% during a first pass and 99.9% if a second was performed. A large selling point for their system is it is “plug-and-play” nature, which means it can easily be integrated with existing sorting infrastructure by using components such as controlled air jets at the end of conveyor belts.^80^ Although this offers a very attractive solution from an economical point of view, it is not yet clear whether such a design would be capable of separating out multiple photoluminescent labels using a single light source like in the other small-scale examples.

### 5.2 Implementation and Life Cycle Assessment

Beyond the practical aspects of sorting plastic waste via photoluminescent labeling, the feasibility of industrial-scale implementation and the resulting economical/environmental benefits must also be considered. Several articles by Woidasky and Lang-Koetz et al. aim to address some of these important questions,^81−83^ specifically in the context of the German recycling sector where very high recycling targets are legislated (63% as of 2022).^84^ However, as photoluminescence-based sorting is still a new pilot-scale technology, most of their data can only be taken as indicative at this stage. They report some discussion with industrial collaborators to identify the main challenges facing the implementation of photoluminescent sorting technology.^81^ In brief, one of the main challenges highlighted was the complex makeup of the packaging value chain with many stakeholders and actors involved, including producers, brand owners, and recyclers. With this complexity comes the need for a universal approach to be agreed upon between all actors.

In terms of environmental impact, life cycle assessment (LCA) is the standard method for gauging a product or process’s impact over its lifetime.^85^ Schwarz et al. used LCA to gauge the environmental performance of plastic recycling and determined that optimal performance can only be achieved with effective pretreatment, such as efficient sorting.^86^ In support of this conclusion, Kusch et al. applied LCA to the results of pilot-scale data for a photoluminescent-based sorting approach of lightweight polyolefin packaging.^82^ They calculated that inclusion of this advanced sorting technology could lead to a carbon reduction of between 578 to 1227 kg of CO_2_ equiv/Mg, depending on the implementation scenario.

Throughout their assessments, Woidasky and Lang-Koetz et al. use two general scenarios, “light” and “complete.”^83^ The “complete” case considers photoluminescent labeling as an entirely new one-step system of sorting plastic waste—an alternative to the current flotation or NIR detection methods. This offers the greatest carbon saving and environmental benefit, with extensive fractionation of the waste stream possible and high reliability. Alternatively, the “light” case considers photoluminescent sorting as an add-on to existing sorting methods, whereby photoluminescence is induced by existing NIR light sources. This offers moderate carbon reduction (578 kg of CO_2_ equiv/Mg) but fails to compete with the “complete” scenario. Despite this, the “light” option was considered to be more attractive because of its ease of implementation with existing infrastructure.^81^ Though this is clearly advantageous for a developed nation like Germany, the “complete” scenario may be a more compelling option for new facilities in the developing world.

## 6 Digital Markers

It should also be mentioned here that alternatives to photoluminescent chemical labels are also being actively explored as a means to improve the sorting of plastic waste streams. Many of these alternate labels involve the use of unique digital tags to separate plastics on an item-by-item basis, rather than categorizing plastics on their polymer type or production batch. A key leader in this technology is the HolyGrail 2.0 project, which is a collaborative initiative between numerous different companies and organizations.^87^ Their technology uses unique barcode-like printed watermarks on packaging to identify waste items, thereby relying instead on high-resolution cameras to capture the code rather than detecting specific spectral emissions. Despite the higher technological demand for this kind of system, promising results have already been reported in European small-scale industrial trials.^88^

Other similar approaches involve embedding data into waste collection in the form of radio frequency ID (RFID) tags, though their impact on cost and recyclability is still in question.^89−91^ Plastic items with any such data-carrying technology included tend to fall into the broader category of “smart” or “intelligent” packaging and can be reviewed in detail elsewhere.^92,93^

## 7 Conclusions and Outlook

The concept of adding photoluminescent markers into plastics to improve waste sorting has now been around for some decades, with gradual developments made in the years since. However, newfound pressures to address the scale of global plastic pollution have renewed and accelerated research interest in this area, with encouraging results already starting to emerge at the pilot-plant scale. These trials, along with supporting LCA, demonstrate the ability of this labeling approach to rapidly yield high-purity waste streams and reduce the environmental impact of plastic waste. However, the exact approach to this new method of waste sorting still needs to be standardized and implemented across the whole plastic value chain with active participation of all its actors.

In this review we have highlighted the broad range of luminophores that may be applied to plastics to identify them in an automated sorting process. We have also highlighted that even relying on a polymer’s own photoluminescence may provide a sorting solution. The prospective markers vary in chemical composition and the required excitation source, and each carries with it particular benefits and drawbacks, as discussed. A similar conclusion can be made for the different approaches to incorporate the markers into plastic products, with each method providing their own unique advantages. As a result, there is no clear “winning” formula to provide the optimal photoluminescent sorting solution, with success likely to be determined on the basis of the specific makeup, use, and end-of-life collection of the target plastic item. Because of the plethora of uses of plastics within modern society, it would appear unlikely that a universal approach could be applied successfully to every plastic-containing product everywhere. Instead, photoluminescent labeling could be best utilized in the recovery of especially valuable polymers or for those products used in high volume, such as in packaging or construction.

A common problem observed across the literature was the need to be highly selective of the photoluminescent marker used on the basis of the existing additives within the plastic, particularly colorants. Black-colored plastics presented the largest issue in this regard and still remain a nuisance for waste sorting. The potentially more expensive options for advanced sorting, such as X-ray fluorescent markers, can offer a robust solution to this problem, but it would be highly advantageous to limit the use of excess additives and colorants in plastics if photoluminescent marking is to be more widely adopted in the future.

IR-excited markers are a particularly attractive solution to enhanced sorting because of their tolerance of other photoluminescent impurities, variety of emission wavelengths, and easy adoption into waste sorting infrastructure, provided that they can be detected using current NIR light sources. Future research into these markers should focus on determining whether these markers can be detected reliably with minimal changes required to existing sorting infrastructure to ensure their economic viability. The use of CQDs is also particularly interesting for photoluminescent-based sorting. Their simple chemical makeup (only carbon) could make them appealing in applications where metal-based additives are not acceptable. However, more study is needed to show if these nanomaterials can be incorporated into polymers using scalable manufacturing methods, such as extrusion, instead of the currently presented solvent-based methods.

A key gap in current literature has been found to be the study of extrusion conditions to integrate photoluminescent materials into different polymer resins, with only one such example found. Further investigation in this area should be a priority, if the extrusion incorporation approach is to be used at a significant scale, to ensure homogeneity of the markers is achieved across various large-volume polymer batches.

Overall, the use of photoluminescent labels to improve waste sorting is a rapidly developing solution to the world’s plastic pollution problem, with industrial-scale advancements being quickly driven by new legislative and social pressures. This new enhanced approach to waste sorting shows great potential to improve both the quantity and quality of recycled plastic materials. This is of major significance toward enabling the future circularity of plastics as larger volumes of high-quality recycled material are demanded. A key challenge in the coming years will be agreeing on a common approach to the wider use of such a sorting technology across all the industry stakeholders. Ideally, any such approach taken should be as accessible as possible to not only developed countries but also the developing world to maximize its global environmental benefit.
